# Simulation-Based Training in High-Quality Cardiopulmonary Resuscitation Among Neonatal Intensive Care Unit Providers

**DOI:** 10.3389/fped.2022.808992

**Published:** 2022-03-09

**Authors:** Pratik Parikh, Ravi Samraj, Henry Ogbeifun, Lydia Sumbel, Kelli Brimager, Mohammed Alhendy, James McElroy, Dottie Whitt, Cody Henderson, Utpal Bhalala

**Affiliations:** ^1^The Children's Hospital of San Antonio, San Antonio, TX, United States; ^2^Driscoll Children's Hospital, Corpus Christi, TX, United States; ^3^Department of Anesthesiology and Critical Care Medicine, University of Texas Medical Branch, Galveston, TX, United States; ^4^Department of Pediatrics, Texas A&M University, College Station, TX, United States

**Keywords:** CPR—cardiopulmonary resuscitation, NICU—neonatal ICU, simulation, quality improvement, debriefing, chest compression quality

## Abstract

**Introduction:**

American Heart Association guidelines recommend the use of feedback devices for CPR provider resuscitation training. There is paucity of published literature regarding the utility of these devices especially in neonates and infants. We sought to evaluate if simulation-based education and debriefing using a CPR feedback device would improve CPR performance on an infant manikin in a cohort of NICU nurses as evaluated by CPR feedback device.

**Methods:**

We conducted a prospective, observational simulation study to assess the quality of chest compressions by NICU nurses before and after debriefing using CPR quality data captured by an accelerometer-based device. Chest compression (CC) depth, rate, recoil, CC fraction and nursing confidence level related to performing a high-quality CPR were compared before and after debriefing using paired *t*-test and Wilcoxon rank sum test.

**Results:**

A total of 62 NICU nurses participated in the study and all of them were Neonatal Resuscitation Program (NRP) certified. There was a significant improvement in CC depth and CC fraction [mean + SD values = 0.79 in + 0.17 (pre-debrief), 0.86 in + 0.21 (post-debrief) (*p* = 0.034) and 56.8% + 17.7 (pre-debrief), 70.8% + 18.4 (post-debrief) (0.0014), respectively]. There was no difference in CC rate (*p* = 0.36) and recoil (*p* = 0.25) between pre and post structured debriefing. The confidence level of nurses in all CPR dynamics (appropriate CC rate, CC depth, team communication, minimizing interruption in CC and coordinating CC with ventilation) was significantly higher after simulation and structured debriefing. All the nurses used 3:1 compression: ventilation ratio of NRP despite the patient being a 4 month old premature baby in the NICU.

**Conclusions:**

Simulation training and debriefing of NICU nurses using CPR feedback device improved their chest compression quality on an infant mannequin and their confidence level for performing high-quality CPR. NICU providers tend to use NRP protocol of 3:1 compression: ventilation ratio during CPR in the NICU irrespective of age of the infant.

## Introduction

The incidence of in-hospital cardiac arrest in infants and children is estimated to be more than 15,000 per year in the United States (1). This major public health burden has been increasing in adults as well as in children (1). Although the rate of survival in pediatric in-hospital cardiac arrest is better than out-of-hospital cardiac arrest (OHCA) (41.1% vs. 11.4%) (2), there is a significant room for improvement, especially for long-term neurologic outcome. High-quality cardiopulmonary resuscitation (CPR) is the foundation of resuscitation (3, 4) and is vital for patient survival and good neurologic recovery (5). Providing adequate chest compression (CC) rate and depth, minimizing CPR interruptions, allowing full chest recoil between compressions, and avoiding excessive ventilations are key components of high-quality CPR (3). High-quality CPR has been shown to improve patient outcomes (6), but CPR quality frequently does not meet standards as recommended by current guidelines, even when performed by well-trained hospital staff (7).

Cardiac arrest in newborns is not as common as in adults. The incidence of cardiopulmonary compromise needing intensive respiratory resuscitative measures and/or CCs to restore cardiopulmonary function is estimated to be <1% of newborns (8). The incidence of cardiac arrest in quaternary neonatal intensive care units (NICUs) has been reported to be ~1–3% of all admissions (9–13). In general, real-life experience of NICU providers with CPR, especially CCs during cardiac arrest events within the NICU, is scarce. To maintain provider competency in high-quality CPR, simulation-based education and debriefing have been used to train healthcare personnel. Simulation can be an effective tool to facilitate the acquisition and maintenance of the cognitive, technical, and behavioral skills (14) and allows for the consolidation of theoretical knowledge and practical skills in a risk-free environment (15).

In addition to simulation-based education and debriefing, CPR feedback devices have been developed to improve the consistency and quality of CCs (16). The first in-hospital randomized controlled trial of CPR feedback device (Cardio First Angel) in cardiac arrest was conducted by Vahedian-Azimi et al. (5). The authors found that the use of the device improved adherence to published CPR guidelines and CPR quality, and it was associated with increased rates of return of spontaneous circulation (ROSC) (5). The American Heart Association (AHA) guidelines recommend the use of feedback devices for CPR provider resuscitation training (17). Several feedback devices are commercially available to assist CPR providers; however, there is paucity of published literature regarding the utility of these devices especially in neonates and infants. In a simulation study using adolescent and infant mannequins, Wagner et al. found that CC quality improved significantly with both visual and verbal feedback compared with instructor-led feedback. They concluded that feedback devices should be implemented during pediatric resuscitation training to improve resuscitation performance (18). To the best of our knowledge, there are no published studies evaluating the utility of CPR feedback devices in neonatal resuscitation. Our study was designed to evaluate if simulation-based education and debriefing using a CPR feedback device would improve CPR performance on an infant mannequin in a cohort of NICU nurses as evaluated by CPR feedback device. We hypothesized that providing simulation-based feedback and debriefing using the data captured on the CPR feedback device would improve the quality of CPR in the study cohort.

## Methods

### Study Design

This single-center, prospective observational simulation study was conducted at The Children's Hospital of San Antonio in August 2019. The study was approved by Baylor College of Medicine Institutional Review Board and Feasibility Committee of Voelcker Clinical Research Center of The Children's Hospital of San Antonio.

### The Primary Outcome Measure

The primary outcome measure was quality of CCs, specifically CC rate, depth, recoil, and CC fraction (CCF). CCF was defined as fraction of total cardiac arrest time when providers were on the chest delivering CCs. As per the AHA guidelines, CCs that were (a) at a rate of 100 to 120 per minute, (b) at a depth equivalent to at least one-third diameter of the chest, (c) with full recoil, and (d) with minimum interruptions (CCF ≥85%) were categorized as high-quality CCs (3).

### Secondary Outcome Measures

Participants' perspective/confidence about delivering high-quality CCs before and after simulation-based training and debriefing and whether any participant-related factor was associated with poor-quality CCs at baseline prior to debriefing were the secondary outcome measures.

### Study Population

The nurses working in our NICU were recruited for the study. A written informed consent was obtained from each study subject. In order to minimize selection bias, we recruited NICU nurses who work either day or night shifts. As there are differences in culture of each NICU, similarly there might be differences in culture of NICU nurses working during different shifts. Thus, to remove any bias, nurses from each shift were recruited. We recruited all the subjects during annual nurse competency sessions organized over a short span of 15 days in August 2019 and asked each subject to sign a confidentiality agreement while signing the consent form. Our annual nurse competency sessions were simulation-based, hands-on training sessions aimed at teaching new skills and/or improving skills related to pediatric acute care management. Being conducted for all our nurses over a relatively short span of time each year, these sessions allowed recruitment of nurses for simulation-based studies and minimized sources of bias related to change in practice over time. Each scenario was sufficiently long to allow team members to perform CCs both before and after debrief, and therefore, the possibility of provider-related bias was minimized. All the simulation conditions including the mannequin used and position of defibrillator pad over the mannequin sternum were uniform for the mock codes, especially before and after debrief.

### Baseline Characteristic Survey

Prior to simulation session, each study participant was asked to fill out demographic data on training level, years in practice, primary location of work, basic and advanced life support certification details, and information on their experience with the actual and mock resuscitation.

## Equipment and Environment

**Figure 1:**
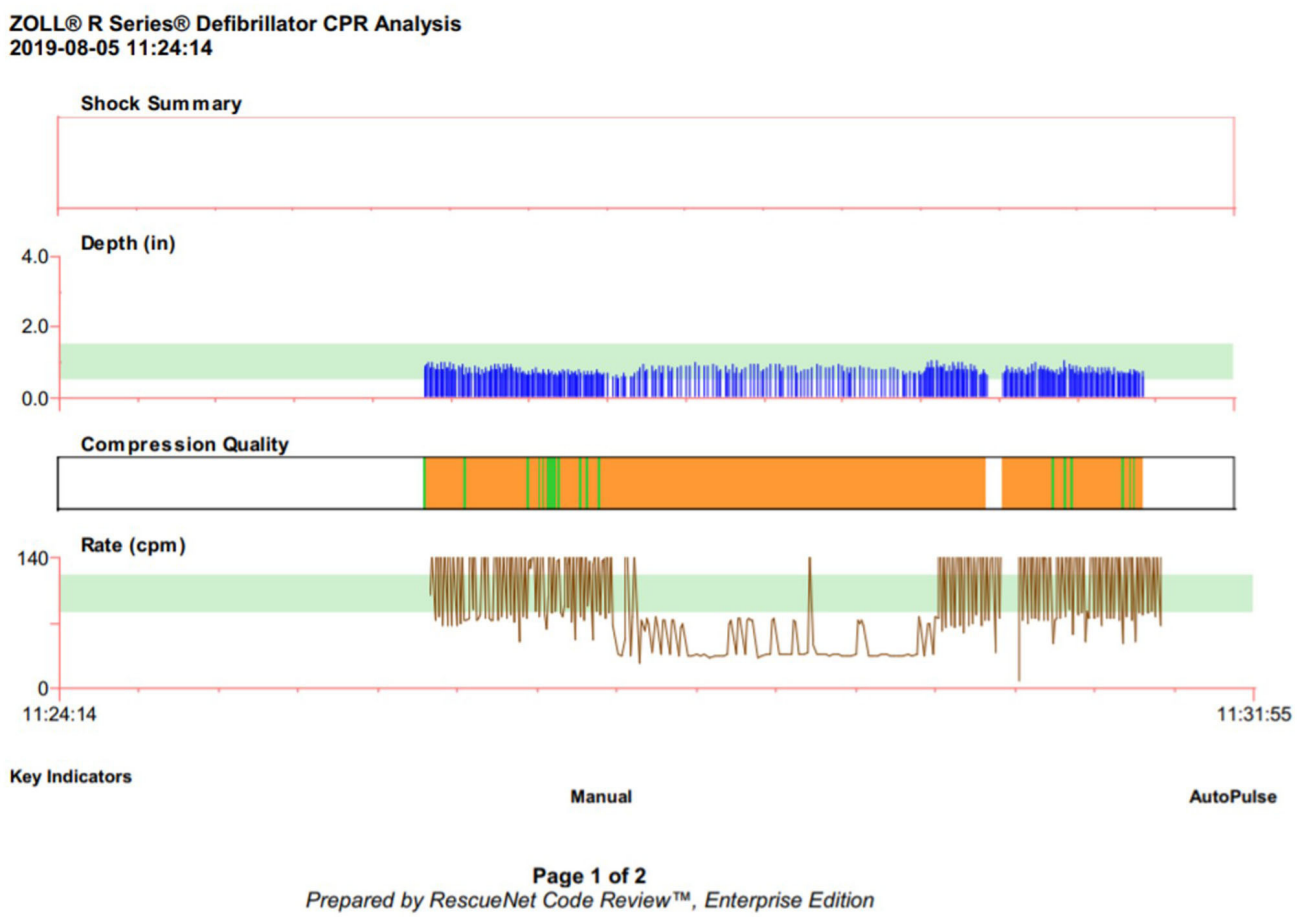
Display of RescueNet CPR quality data for debriefing.

The code scenario was conducted at the simulation laboratory at our institution. A preprogrammed infant simulator mannequin (SimBaby™; Laerdal Medical, USA) with an anterior–posterior chest diameter of 3 inches was used in our study for all the simulation sessions. The mannequin was placed on a firm surface of the neonatal warmer at the same height for all the scenarios. The entire setup, including the positioning and placement of the neonatal mannequin on the neonatal warmer, was uniform during each scenario. A step stool was provided to the participants upon request. Full simulator functions, including vital sign monitoring, audiovisual feedback, breath sounds, chest rise, heart sounds, and palpable pulses, were activated to achieve a high level of realistic stimulation. Accelerometer-based CPR feedback device (R Series; ZOLL Medical, Chelmsford, MA, USA) was used to capture the quality of the CPR during the simulation codes. Pediatric-type pads were used for data collection. The data collected from the device included CC rate, depth, recoil, and CCF. The ZOLL RescueNet Code Review software (ZOLL Medical) was used to analyze the CC data and conduct data-driven code debriefing (Figure 1).

### Simulated Pulseless Electrical Activity Cardiac Arrest Scenario and Debrief

As one of the objectives of the study was to assess if the NICU nurses would use the Neonatal Resuscitation Program (NRP) vs. the Pediatric Advanced Life Support (PALS) guidelines for cardiac arrest in an infant managed in the NICU, we chose a scenario of respiratory arrest progressing into a cardiac arrest in a 4-month-old, ex 34-week premature baby in the NICU. During prebrief, all the study participants were informed that each participant was required to perform CCs during each scenario before and after debriefing. The participants were described the case scenario^1^ and asked to deliver CCs on top of the ZOLL pads applied over the sternum of the mannequin. The participants were blinded to CC quality during the resuscitation. In each scenario, there were enough team players for supporting cardiopulmonary function, including ventilation and CCs. As the team players rotated the roles, each nurse performed CC during the scenario. A structured, 30-min-long debrief was conducted soon after the session focusing on CPR, teamwork, and communication. During debriefing, we used RescueNet data on the quality of compressions to acknowledge gaps in performance related to compressions and then demonstrated CCs on the mannequin. In order to avoid any bias, the same instructor (U.B.), who had an extensive training, experience, and expertise with a structured debrief, conducted the debriefing for all the simulation sessions. Using the CC quality report generated by the ZOLL RescueNet Code Review software, the participants were debriefed specifically about the CC quality. Soon after debrief, all the participants were asked to perform CCs again with the ZOLL pads over the sternum. During the postdebrief simulation session, the participants remained blinded to just-in-time feedback on the quality of CCs. The participants' hands-on CC skills before and after debriefing were assessed and compared using the ZOLL RescueNet Code Review software. To evaluate the confidence of the participants in various complex CPR dynamics, we conducted a survey among study participants before and after simulation debriefing. We used a standard, validated Likert scale to obtain objective information about their confidence level related to CPR quality and teamwork.

### Data Abstraction

Each scenario was captured on a video, and video files were transferred to and stored in a secured, password-protected, institutional file-sharing tool called Baylor College of Medicine Box (Box, Inc., Redwood City, CA, USA). Two independent reviewers (H.O. and L.S.) evaluated each video file, and the moderator (U.B.) resolved any disagreement on the findings of the two reviewers. During video evaluation of each scenario, the reviewers assessed for the ventilation–compression ratio to determine if the providers followed the NRP or PALS guidelines for resuscitation. Data were entered into a Microsoft Excel^®^ database and analyzed using GraphPad Prism (GraphPad Software, Inc., San Diego, CA, USA).

### Statistical Analysis

All statistical analyses were done in GraphPad Prism (GraphPad Software, Inc.). Analyses were conducted for baseline characteristics and outcome variables. For categorical variables, proportions were compared using the χ^2^ test. The variables not normally distributed were reported in median with interquartile range (IQR). Median and IQR were determined for CC rate, depth, recoil, and CCF for each group of participants. Paired *t*-test was used for comparative study of the predebriefing and postdebriefing CC rate, depth, recoil, and CCF. Wilcoxon rank sum test was used to compare the presimulation and postsimulation nursing survey. In all analyses, *p* < 0.05 was considered significant.

## Results

A total of 62 NICU nurses participated in the study. Of the study participants, 100% were NRP certified, 77.6% of nurses were Basic Life Support (BLS) certified, and 8.6% of nurses were PALS certified. The participants also varied in their neonatal nursing experience: 16% of nurses had more than 20 years of experience, 31% with 10–20 years of experience, 27% with 5–10 years of experience, and 26% of nurses had <5 years of experience.

**Figure 2:**
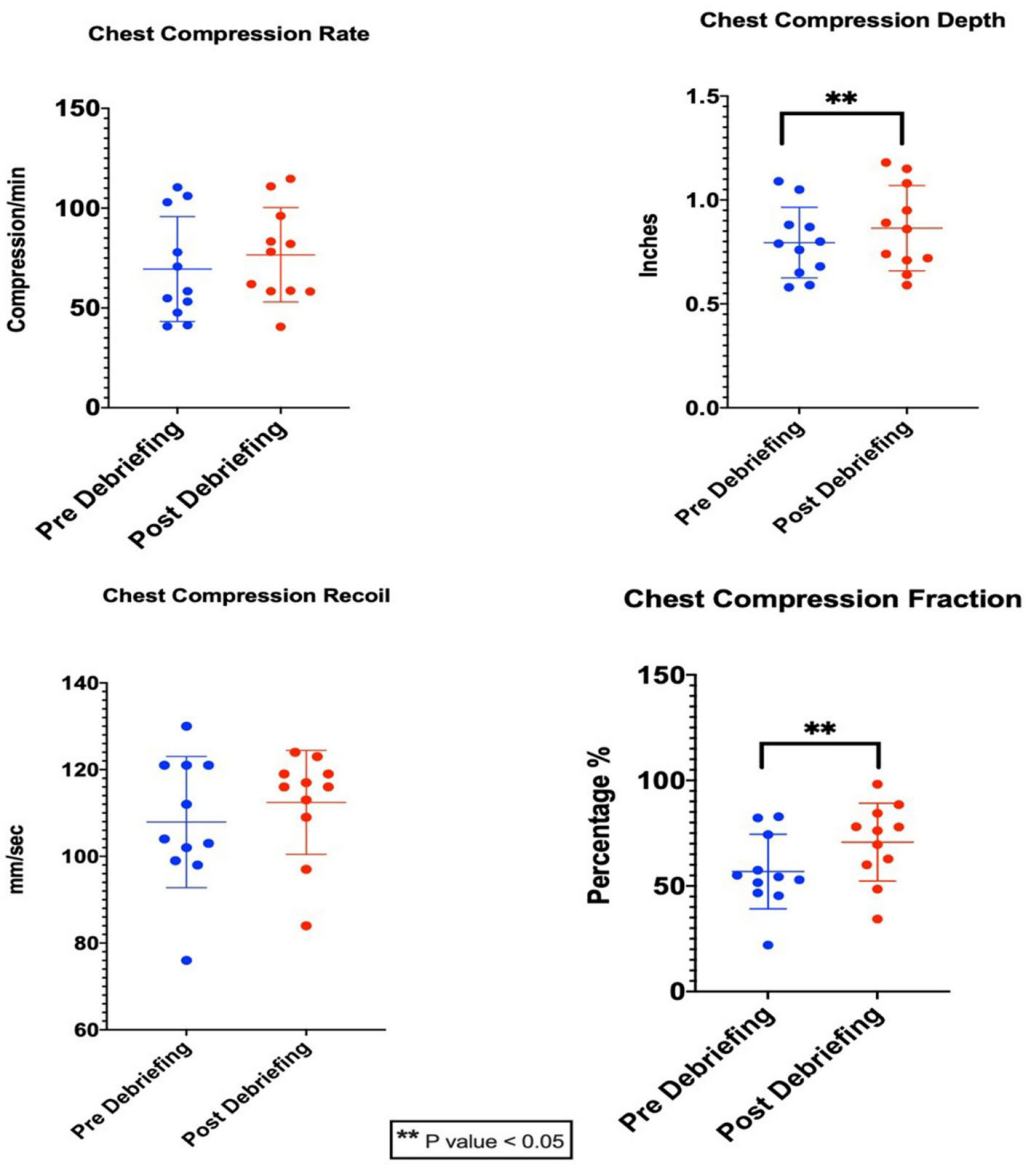
Quality parameters of chest compression between predebriefing and postdebriefing during NICU CPR.

There were 11 scenarios each before and after debriefing. In each scenario, there were average six participants. Each scenario was sufficiently long to allow team members to perform CCs both before and after debrief. The CC rate, depth, CC recoil velocity (CCRV), and fraction (mean ± SD values) for predebriefing and postdebriefing were 69.5 ± 26.3/min, 76.6 ± 23.6/min, 0.79 ± 0.17 in, 0.86 ± 0.21 in, 107.9 ± 15.12 mm/s, 112.5 ± 12 mm/s, and 56.8 ± 17.7%, and 70.85 ± 18.4%, respectively (Table 1). There was a significant improvement in CC depth (*p* = 0.034) and CCF (0.0014) after structured debriefing. There was no difference in CC rate (*p* = 0.36) and recoil (*p* = 0.25) between pre- and post-structured debriefing (Figure 2).

**Table 1:** Comparison of quality parameters of chest compression between predebriefing and postdebriefing during neonatal CPR simulation.

**Quality parameters**	**Predebriefing (*n =* 62)**	**Postdebriefing (*n =* 62)**	***p*-value**
Compression rate (compressions per minute)	69.5 ± 26.3	76.6 ± 23.6	0.36
Compression depth (inches)	0.79 ± 0.17	0.86 ± 0.21	0.034^*^
Compression recoil (mm/s)	107.9 ± 15.12	112.5 ± 12	0.25
Compression fraction (%)	56.8 ± 17.7	70.85 ± 18.4	0.0014^*^

* *p < 0.05*.

**Figure 3:**
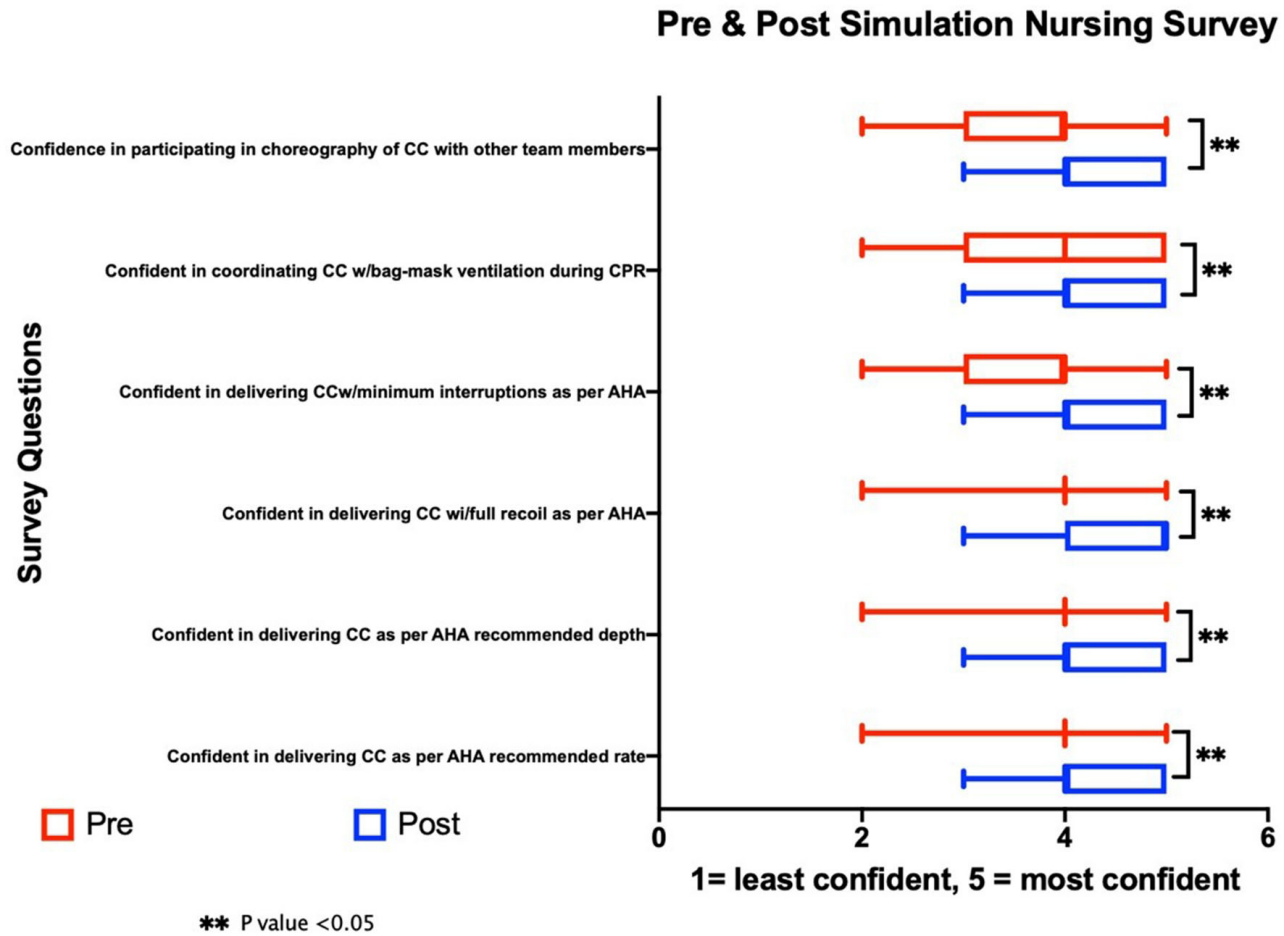
Comparison of presimulation and postsimulation nursing survey.

Our survey found that the confidence level of nurses in all CPR dynamics (appropriate CC rate, CC depth, team communication, minimizing interruption in CC, and coordinating CC with ventilation) was significantly higher after simulation and structured debriefing (Figure 3). This difference was most profound in participants with no real-life CPR experience, whereas there was no significant difference in experienced participants with previous real-life CPR exposure. Similar to delivery room resuscitation, all the nurses used 3:1 compression-to-ventilation ratio of NRP despite the patient being a 4-month-old premature baby in the NICU.

## Discussion

Simulation is defined as a technique used to replace or amplify real experiences with guided experiences that evoke or replace substantial aspects of the real world in a fully interactive manner (19). Resuscitation training programs routinely use simulation and debriefing for training modality for their CPR providers. Studies have shown that debriefing following simulation-based education can improve the quality of CPR and are associated with increased ROSC (20, 21). Our study evaluated if simulation-based education and debriefing using an accelerometer-based CPR feedback device resulted in improvement in CPR quality metrics among NICU nurses. The study demonstrated a significant improvement in CC depth and fraction after simulation-based training. The study also demonstrated a significant improvement in the confidence level of NICU nurses in delivering high-quality CCs. We found that following simulation-based education and debriefing, there was a significant improvement in CC depth (*p* = 0.019) and CCF (*p* = 0.00293), but there was no significant improvement in CC rate/recoil.

Several CPR adjunct devices have been developed and are commercially available to assist in delivering high-quality CPR (22). Previously published studies have reported improved CPR quality, specifically CC rate and depth using CPR feedback devices (23–27). Most of these studies used technology to provide real-time feedback or prompts related to quality of CPR to evaluate the CPR performance (26), and only one study, that by Dine et al., reported their findings of improved CPR performance following real-time feedback and/or debriefing (27). Our study focused on CC performance in an infant mannequin following debriefing using objective data on CPR performance. In our study, we used ZOLL^®^ R Series^®^, a monitor/defibrillator that provides real-time audio and visual feedback on CPR quality measures. It provides numeric displays of CC depth and rate and visual indicators of compression release. The device uses accelerometer detection technology with detection limits for CC depth between 0.75 in (1.9 cm) and 3.0 in (7.6 cm), with an accuracy of ±0.25 inches (0.6 cm) and for CC rate between 50 and 150 compressions per minute.

Current neonatal resuscitation guidelines published in 2020 recommend initiating CPR when the heart rate is <60 beats/min with a ratio of three compressions before and after each ventilation (3:1 for 120 total events per minute)(28), whereas the recommended compression: ventilation ratio for infants is 15:2 when two providers are available or 30:2 when only one provider is available (3). All participants in our study followed the 3:1 compression-to-ventilation ratio for the NICU CPR scenario in an infant who was past the neonatal period and beyond cardiopulmonary physiologic transitions of postneonatal period. This is probably secondary to their training and working in the neonatal unit where NRP guidelines are followed. These findings corroborate with the results of the survey of NICU providers related to resuscitation practices for infants in the NICU (29). Animal studies have shown similar times to ROSC and mortality rates with alternative compression-to-ventilation ratios (30). The two-thumb technique for CCs has been demonstrated to be superior to the two-finger technique in achieving greater depth and less intercompression variability (31). All our study participants uniformly used the two-thumb technique during CPR in our sessions.

A lower CC rate will result in lower perfusion pressure, whereas a faster rate causes compressor fatigue resulting in eventual CC quality deterioration. Similarly, CC performed at an inappropriate depth and incomplete chest relaxation result in reduction of perfusion pressure, leading to lower changes of recovery from cardiac arrest (32). Abella et al., in their in-hospital observational study using a feedback device, found that CC rates were below published resuscitation recommendations, and suboptimal compression rates in their study correlated with poor ROSC (33). CC rate in our study was lower than recommended in the predebriefing group (69.5 ± 26.3/min). Although the number improved after debriefing (76.6 ± 23.6/min), it was statistically not significant (*p* = 0.36) and still below the recommended target rate. We believe that the low CC rate observed in our study could be related to multiple factors such as a lack of use of metronome or feedback system to achieve recommended CC rate and stiff chest wall of mannequin causing provider fatigue and potentially related to interruptions in CC to allow airway management, endotracheal intubation, and switching of the team roles. Further education, continuing simulation sessions, and simultaneous use of audiovisual feedback devices during CPR may help in achieving the target CC rate in CPR providers.

Complete and fast chest recoil during CPR ensures coronary perfusion pressure and myocardial blood flow (33). In a study conducted on 981 OHCAs in adult patients, fast CCRV ≥400 mm/s was associated with increased survival and improved favorable neurologic outcome compared with slower CCRV (34). In addition, the authors found that there was a 5.2% increase in the adjusted odds of survival for each 10-mm/s increase in CCRV. In our study, the CCRV was similar in both groups (107.9 ± 15.12 mm/s in the predebriefing group, whereas it was 112.5 ± 12 mm/s in the postdebriefing group, *p* = 0.25). This was expected not to change as we did not use a feedback device displaying CCRV during CPR to help the participants improve this metric. We only used the data captured on the feedback device during the debriefing after the code. CCRV in our study was lower than the previously published fast CCRV associated with better outcomes (33). It is difficult to compare the CCRV in mannequin simulations to the CCRV in real patients, as the physical characteristics of chest wall and recoil are different. Moreover, optimal CCRV may be different in children and neonates compared with adults because of the difference in body habitus and elasticity of the chest wall. Further studies in elucidating the ideal CCRV in mannequins would help in strengthening the feedback to CPR providers during simulation and increase the quality of eventual CPR delivered.

Pediatric BLS recommends a compression depth of approximately one-third of the anterior–posterior diameter of the chest in infants and neonates (35). For most infants, this is 1.5 inches (4 cm). AHA recommends an upper limit for compression depth of not more than 2.4 inches; however, there are no recommendations for the upper limit of compression depth in children. In our cohort, we found a compression depth of 0.79 in ±0.17; following debriefing, there was a statistically significant improvement to 0.86 in ±0.21. Although we saw a significant improvement in CC depth following debriefing, overall, an increase in CC depth of only 9.2% may or may not be clinically relevant in a neonate/infant and therefore needs further evaluation in clinical studies. There are inherent limitations of mannequins especially in relation to chest wall shape, compliance, and other characteristics as compared with human chest. It is particularly pertinent in case of neonates and infants—the chest wall in a human neonate is much more compliant than the chest wall in a neonate/infant mannequin. It is likely that the low CC depth achieved by providers in our study which used an infant mannequin was related to stiff chest wall of the mannequin. It is important to understand if simulation-based training could improve performance at the bedside and eventually improve clinical outcome. The primary aim of our study was to assess provider performance and confidence in simulated scenario, and future studies are needed to assess the effects on clinical outcome.

Another metric, CCF, has been used to evaluate the quality of CPR. This metric is designed to study the interruptions in CCs. The CCF is defined as the proportion of resuscitation time without spontaneous circulation during which CCs were administered. A study in OHCA patients with ventricular fibrillation found that an increased CCF is independently predictive of better survival in patients who experience a prehospital ventricular fibrillation/tachycardia cardiac arrest (36). An international resuscitation collaborative in their study of 112 events of in-hospital pediatric cardiac arrest found a median CCF of 0.94 (0.79–1.00). In our study, we found a CCF of 56.8% ± 17.7% before debriefing, which increased to 70.85 ± 18.4% following debriefing. Although the previous guidelines were to achieve a CCF of 80% (37), AHA CPR guidelines 2020 suggest that it is reasonable to target a CCF of at least 60% (38). Our predebriefing CCF was below the recommended value, which improved to reach the AHA recommended target levels after debriefing.

To evaluate the confidence of the participants in various complex CPR dynamics, we conducted a survey among study participants before and after simulation debriefing. Our findings showed that simulation debriefing significantly improved the confidence of participants not only in CPR metrics but also in complex CPR dynamics such as team communication. Although we did not assess the effects of human factors such as team practice and training, stress, cognitive workload, communication, and so on, on CPR performance, we plan to use video recordings of the scenario to assess human factors and CPR performance.

## Limitations

There were some limitations to our study. First, our study included a small group of participants from a single medical center. The participants did not receive just-in-time visual feedback from the device during CPR; however, the study was not designed to evaluate the effect of device feedback on CPR parameters. We plan to conduct further studies in this regard in the future. The use of an accelerometer prevents the assessment of the correct placement of the thumbs over the chest. This could have potentially influenced CC quality. As the simulation conditions, including the use of an accelerometer, did not differ between simulation sessions before and after the debriefing, the CCs before and after debriefing were comparable. As the study was conducted using mock codes in a team setting during the annual nursing competency, the study could not compare CC skills of individual NICU providers before and after debriefing. It is possible that the results could have been different if we compared individual performance vs. the performance by providers in team setting, and it is possible that there was provider-related bias affecting our results. We believe that each mock scenario was sufficiently long to allow team members to perform CCs both before and after debriefing, and therefore, possibility of provider-related bias was minimized. Another source of bias is the Hawthorne effect, based on which the rescuer can change its way of performing the technique by being observed or evaluated. However, this effect was present both during predebriefing and postdebriefing simulation, and therefore, it is appropriate to compare the predebriefing and postdebriefing performance. Although we did not follow up with the providers to assess retention of performance at or beyond 3 months, we plan to study the skills retention at 3 month follow-up in the future. Overall, the goal of simulation is to positively impact the skill performed, to improve skills during routine clinical practice. Lastly, as the NICU nurses are not generally experienced with delivering CCs on top of the defibrillator pad placed over the sternum, it is possible that it contributed to their CC performance. As all the simulation conditions including the mannequin used and position of defibrillator pad over the mannequin sternum were uniform for the mock codes, especially before and after debriefing, we believe that mannequin- and defibrillator pad–related issues did not affect the study results. Our study was not designed to compare debriefing vs. CC quality record as two separate interventions, and therefore, we were unable to discern which intervention influenced the CPR performance the most. It would be important to test them separately in the future.

## Conclusion

CPR simulation training and debriefing of participants using a CPR feedback device improved CC quality for simulated NICU CPR scenario in a cohort of NICU nurses in our study. The improvement following debriefing was primarily in CC depth and CCF. In addition, simulation debriefing using CPR feedback device significantly increased the confidence of study participants in delivering high-quality CPR. Our study also showed that the NICU providers tend to use NRP protocol of 3:1 compression-to-ventilation ratio during CPR in the NICU irrespective of age of the infant. Further studies are needed to evaluate the utility of CPR feedback devices in providing just-in-time audiovisual feedback to improve CPR quality as well as to study the optimal CCRV in different-sized mannequins. Future studies are needed to assess if simulation-based training of high-quality CPR among NICU providers could improve outcomes of CPR in neonates and infants.

## Data Availability Statement

The raw data supporting the conclusions of this article will be made available by the authors, without undue reservation.

## Ethics Statement

The studies involving human participants were reviewed and approved by Baylor College of Medicine IRB. The patients/participants provided their written informed consent to participate in this study.

## Author Contributions

All authors listed have made a substantial, direct, and intellectual contribution to the work and approved it for publication.

## Conflict of Interest

The authors declare that the research was conducted in the absence of any commercial or financial relationships that could be construed as a potential conflict of interest.

## Publisher's Note

All claims expressed in this article are solely those of the authors and do not necessarily represent those of their affiliated organizations, or those of the publisher, the editors and the reviewers. Any product that may be evaluated in this article, or claim that may be made by its manufacturer, is not guaranteed or endorsed by the publisher.

## Footnotes

^1^NICU pulseless electrical activity case scenario: A 4-month-old, ex 34-week premature baby with trisomy 21, intrauterine growth retardation, pulmonary hypertension, chronic lung disease, oxygen dependency, and nasogastric feeding tube dependency due to gastroesophageal reflux disease, currently feeding and growing in NICU. A few minutes after his bolus nasogastric tube feed, he develops sudden-onset respiratory distress with progressively worsening desaturation, cyanosis, and bradycardia. The mother at the bedside has called for help and pushed code button at the bedside. You and your team of NICU nurses are now at the bedside to assess his condition and initiate his stabilization. The patient's weight is 4 kg. The patient has bradycardia with heart rate <60 beats per minute, absent peripheral pulses, apnea and unresponsiveness when you arrive at the bedside.
